# NT-proBNP Changes in Patients with Ascites during Large Volume Paracentesis

**DOI:** 10.1155/2013/959474

**Published:** 2013-09-22

**Authors:** Vi Nguyen, Rob Zielinski, Paul Harnett, Katherine Miller, Henry Chan, Nikitha Vootakuru, Priya Acharya, Montaha Khan, Oliver Gibbs, Sarika Gupta, Anjla Devi, Shani Phillips, Jacob George, David van der Poorten

**Affiliations:** ^1^Storr Liver Unit, Westmead Millennium Institute, University of Sydney at Westmead Hospital, Westmead, Sydney, NSW 2145, Australia; ^2^Department of Medical Oncology, Westmead Hospital, Sydney, NSW 2145, Australia; ^3^Department of Medicine, Westmead Hospital, Sydney, NSW 2145, Australia; ^4^Department of Gastroenterology & Hepatology, Westmead Hospital, Sydney, NSW 2145, Australia

## Abstract

*Background*. *N*-terminal probrain natriuretic peptide (NT-proBNP) is a hormone involved in the regulation of cardiovascular homeostasis. Changes in serum NT-proBNP during large volume paracentesis (LVP) in patients with ascites have never before been examined. *Aims*. To determine if significant changes in serum NT-proBNP occur in patients undergoing LVP and the associated clinical correlates in patients with cirrhosis. *Method*. A total of 45 patients with ascites were prospectively recruited. Serum NT-proBNP, biochemistry, and haemodynamics were determined at baseline and at key time points during and after paracentesis. *Results*. 34 patients were analysed; 19 had ascites due to cirrhosis and 15 from malignancy. In those with cirrhosis, NT-proBNP decreased by 77.3 pg/mL at 2 L of drainage and 94.3 pg/mL at the end of paracentesis, compared with an increase of 10.5 pg/mL and 77.2 pg/mL in cancer patients at the same time points (*P* = 0.05 and *P* = 0.03). Only congestive cardiac failure (CCF) was an independent predictor of significant NT-proBNP changes at the end of drainage in cirrhotic patients (*P* < 0.01). There were no significant changes in haemodynamics or renal biochemistry for either group. *Conclusion*. Significant reductions in serum NT-proBNP during LVP occur in patients with cirrhosis but not malignancy, and only comorbid CCF appeared to predict such changes.

## 1. Introduction

Ascites is the most common cause of hospital admissions in cirrhotic patients. The development of ascites predicts a mortality of approximately 15% and 44% at one and five years, respectively [1]. Total therapeutic large volume paracentesis (LVP) has been shown to be a safe means of treating tense or refractory ascites [2, 3]. Postparacentesis circulatory dysfunction (PCD) describes the development of neurohumoral changes and circulatory disturbances which can arise following fluid shifts induced by LVP in patients with cirrhosis. This syndrome has been associated with a significant increase in intrahepatic vascular resistance, higher rates of ascites recurrence, the development of hepatorenal syndrome, dilutional hyponatraemia, and decreased overall survival [4, 5]. Supplementation with plasma expanders during paracentesis, particularly albumin, can reduce this risk [6, 7]; however, it still occurs in 16–27% of patients [8, 9]. Currently there are no well-defined risk factors that can reliably identify the subset of patients who are at greatest risk of PCD.

Brain natriuretic peptide (BNP) is a hormone produced by the cardiac ventricles which is involved in the regulation of fluid volumes and cardiovascular homeostasis. Elevations in plasma BNP reflect fluid overload, while reductions in BNP reflect relative intravascular depletion. Plasma BNP is increased in a number of disease states including congestive cardiac failure (CCF), chronic renal failure (CRF), hypertension (HT), and cirrhosis [10]. Serum BNP levels have been shown to correlate with more advanced cirrhosis and have been shown to independently predict shorter survival in the pre- and posttransplant setting [11–13]. It is also thought to be a useful marker for the diagnosis of insidious cardiac dysfunction in cirrhotic patients [11, 12]. *N*-terminal- (NT-) proBNP is the prohormone of BNP with a more stable and longer half-life which allows for more reliable assessment of associated changes. Dynamic changes in serum NT-proBNP in cirrhotic patients undergoing LVP have never before been examined.

The primary aim of the current study was to determine whether significant changes in serum NT-proBNP occur in patients with ascites secondary to cirrhosis undergoing LVP in comparison to patients with noncirrhotic ascites. We further sought to determine if changes in serum NT-proBNP during LVP correlate with alterations in haemodynamics or serum biochemistry and whether colloid administration could prevent these changes. We hypothesised that significant serum NT-proBNP changes would occur in the cirrhotic but not the malignant cohort.

## 2. Materials and Methods

From December 2011 to November 2012, consecutive patients with ascites requiring therapeutic paracenteses were prospectively recruited from the South Western Area Health Service at Westmead Public Hospital in Sydney, Australia. Both inpatients and outpatients were included. The study was approved by the hospital ethics committee in accordance with the Helsinki Declaration of 1975, and written and informed consent was obtained from all participants. 

Patients were included if their age was greater than 18 years, the treating physician had deemed a therapeutic paracentesis as an appropriate and safe treatment for the management of ascites, and the patient had not suffered any prior complications from an ascitic drainage which required hospital admission. Participants in the cirrhosis cohort were confirmed either clinically or with biopsy, while those in the malignancy cohort had diagnoses of cancer confirmed either histologically or cytologically. Patients were excluded if they had evidence of concurrent cirrhosis and malignancy (excluding hepatocellular carcinoma) and if any medical or psychiatric illnesses precluded the acquisition of informed consent.

Information on the underlying cause of cirrhosis or type of malignancy, medical comorbidities, other complications of portal hypertension in the cirrhotic patients (namely, varices, previous spontaneous bacterial peritonitis, or hepatic encephalopathy) and the use of diuretics were recorded at recruitment.

All patients underwent paracentesis using a 16-gauge plastic cannula which was inserted under aseptic technique into the lower abdominal cavity. Each cannula was attached to a collection set with drainage into volume-graduated bags. Drainage was continued until no further abdominal fluid could be collected. All patients underwent measurements of heart rate, blood pressure, serum biochemistry, and NT-proBNP at baseline, at 2 litres (L) of drainage, and at the end of drainage. In the group with ascites secondary to cirrhosis 100 mls of 20% albumin (Albumex 20) was also administered for every 2 L of fluid drained, and serum NT-proBNP was measured at thirty minutes following albumin administration after 2 L of drainage. Only patients who drained >3 L of ascitic fluid were considered to have had LVP. A subset of the cirrhotic cohort also had serum biochemistry and haemodynamics remeasured at 5–7 days following paracentesis to examine delayed changes with LVP.

Serum NT-proBNP measurements were made using a two-site (sandwich complex) immunoassay (Elecsys proBNP II, Roche Diagnostics GmbH, Mannheim, Germany) with a cutoff value ≥125 pg/mL considered as abnormal. Biochemical analyses for protein, albumin, lactate dehydrogenase (LDH), and differential cell counts were also undertaken on the ascitic fluid collected in each patient.

Statistical analysis was performed using *SPSS* software (SPSS version 16.0, Chicago, IL, USA). Changes in BNP were assessed by *t*-tests and nonparametric correlation, and two-tailed *P* values of ≤0.05 were regarded as significant throughout. Multivariate analysis on clinical correlates was assessed using backward stepwise regression.

## 3. Results

### 3.1. Baseline Characteristics

A total of 45 patients were recruited during the study period. Of these, only 34 patients who drained >3 L of fluid were included for analysis. Nineteen had ascites secondary to cirrhosis, and fifteen had ascites secondary to malignancy. The patients in each group were similar with respect to age and gender (Table 1). The most common cause of cirrhosis was alcohol (7/19 [37%]), while the commonest cause of malignancy was gynaecological (7/15 [47%]). All cirrhotic patients had either Child-Pugh B or C disease, and the median MELD score was 16.

As expected, patients with cirrhosis had a significantly higher serum bilirubin (58.2 *μ*mol/L versus 11.6 *μ*mol/L, *P* = 0.03), INR (1.56 versus 1.05, *P* < 0.01) and serum albumin to ascitic gradient (18 versus 15, *P* = 0.03) but lower platelet counts (125 × 10^9^/L versus 463 × 10^9^/L, *P* < 0.01) and ascitic fluid protein (25 g/L versus 34 g/L, *P* = 0.02). Cirrhotic patients were also more likely to be on diuretics (12/19 (63%) versus 2/15 (13%), *P* < 0.01) and had slower resting heart rates (79 versus 93 beats per minute, *P* = 0.02).

A greater number of cirrhotic patients had concurrent CCF/CRF/HT (12/19 (63%) versus 3/15 (20%), *P* = 0.01), but when CCF was considered alone, this was not statistically significant (6/19 (32%) versus 1/15 (7%), *P* = 0.06). Mean serum NT-proBNP was not statistically different between the two groups (1191.3 pg/mL in cirrhotics versus 567.3 pg/mL in malignancy, *P* = 0.16); however, in the cirrhotic cohort, those with concurrent CCF/CRF/HT had significantly higher baseline NT-proBNP levels (1724.2 pg/mL versus 277.7 pg/mL, *P* = 0.03).

### 3.2. Changes in Serum NT-proBNP

**Figure 1:**
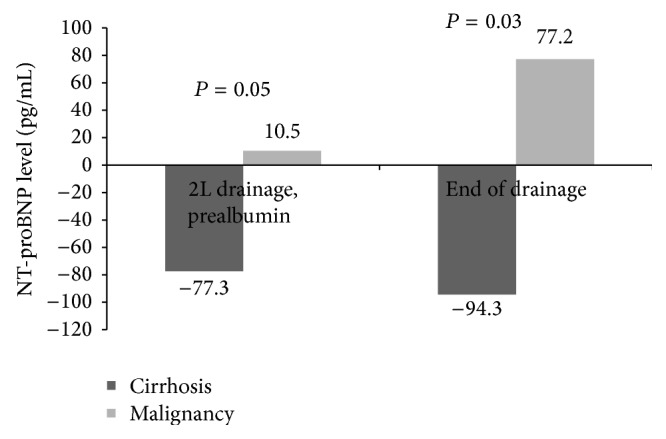
Mean changes in NT-proBNP levels during key LVP time points in each group. Mean NT-proBNP change at 2 L prealbumin −77.3 pg/mL in cirrhotics versus +10.47 pg/mL in cancer patients (*P* = 0.05) and −94.3 pg/mL versus +77.2 pg/mL at the end of drainage (*P* = 0.03). 95% confidence interval −173.7–−1.7 and −323.1–−19.7 in cirrhotics for both time points, respectively.

Serum NT-proBNP levels decreased by 77.3 pg/mL at 2 L of drainage (prealbumin administration) in those with cirrhosis compared to an increase of 10.47 pg/mL in the patients with malignancy (*P* = 0.05, 95% CI: −173.7–−1.7). Similarly, at the end of drainage NT-proBNP decreased by 94.3 pg/mL in the cirrhotics versus an increase of 77.2 pg/mL in the malignancy cohort (*P* = 0.03, 95% CI: −323.1–−19.7) (Figure 1). The changes in serum NT-proBNP levels for the cirrhotic cohort at 2 L of drainage and at the end of drainage in comparison to baseline were both significant (*P* = 0.02 and *P* = 0.05, resp.). 

### 3.3. Clinical Correlates

In the cirrhotic cohort, change in serum NT-proBNP at the end of drainage was significantly associated with comorbid CCF but not CRF or HT (mean decline of 272.5 pg/mL versus 5.17 pg/mL, *P* = 0.04). Higher MELD scores also correlated with significant NT-proBNP changes at the end of drainage but failed to reach statistical significance at 2 L of drainage (*P* = 0.05 and *P* = 0.06, resp.). Conversely, baseline GGT and SAAG appeared to have significant association at 2 L of drainage but not at the end of drainage. On multivariate analysis, only CCF was a predictor of significant NT-proBNP changes at the end of drainage (*P* < 0.01).

The aeitology of liver disease and Child-Pugh score were not significant and neither was a history of gastrooesophageal varices, spontaneous bacterial peritonitis, or hepatic encephalopathy. Current diuretic therapy and the total volume drained also appeared to be unimportant (Table 2).

Changes in haemodynamics and serum biochemistry did not significantly correlate with any changes in serum NT-proBNP at either time point nor were any significant delayed changes seen in the subgroup of patients (*n* = 6) who had these measurements repeated at 5–7 days from paracentesis.

### 3.4. Changes with Colloid Administration

Serum NT-proBNP levels did not significantly change from 2 L of ascites removal to the end of drainage (mean decline of 17 pg/mL, *P* = 0.67). This corresponded with the administration of 185 mls of concentrated albumin for a mean 3.7 L of additional fluid removed. Similarly, in the subset of patients with concurrent CCF/CRF/HT (*n* = 12), NT-proBNP levels were not significantly altered from 2 L of drainage to the end of paracentesis (mean decline of 41.7 pg/mL, *P* = 0.32), corresponding to a mean additional drainage of 4.1 L with 205 mls of supplemental albumin (Table 3).

## 4. Discussion

To our knowledge, this is the first study examining dynamic changes in serum NT-proBNP levels during large volume paracentesis (LVP). Our results indicate that (i) significant serum NT-proBNP changes occur during LVP in patients with cirrhosis but not malignancy, (ii) cirrhotic patients with CCF show greatest reductions in serum NT-proBNP and may therefore be at greatest risk of haemodynamic complications following LVP, and (iii) albumin supplementation appears to ameliorate the intravascular depletion associated with LVP.

As anticipated, significant NT-proBNP reductions were not seen in the malignant cohort during LVP as the pathogenetic mechanisms leading to ascites in these patients are thought to relate to lymphatic obstruction and increased capillary permeability rather than cardiovascular dysfunction [14]. Results from three patients with concurrent cirrhosis and limited HCC were included in the analysis, with their serum NT-proBNP changes not significantly different from those seen among cirrhotic patients without HCC. Despite the greater number of patients with concurrent CCF/CRF/HT in the cirrhotic cohort, when CCF was considered alone, this was not statistically different between the two groups. CRF and HT also did not correlate with more significant changes in serum NT-proBNP when examined separately. Although the small patient numbers limit the statistical reliability of our results, these findings would support the hypothesis that inherent aberrant neurohumoral and cardiovascular alterations precede the development of ascites in patients with advanced cirrhosis, and this is what places them at risk of intravascular changes during LVP [15, 16]. 

Our findings of significant NT-proBNP changes at all measured time points during LVP suggest that relative intravascular depletion occurs relatively early during LVP and even with small volumes of fluid removal (i.e., at 2-3 L). The clinical significance of such changes may be questioned given that they did not seem to correlate with any gross changes in haemodynamic parameters nor in biochemistry either at the end of drainage or at one week after paracentesis (when PCD is most apparent). However, it is likely that the liberal administration of albumin may have attenuated any potentially significant clinical changes seen.

Although it remains somewhat controversial regarding the issues of when, how, and how much colloid should be administered during LVP, it has been recommended that 6–8 g/L of albumin should be administered following the removal of volumes greater than 4-5 L in order to minimise the risk of PCD [17, 18]. Albumin supplementation is often recommended to begin following the end of drainage, when the haemodynamic shifts are thought to begin. It was our practice in the current study to administer 10 g/L of albumin, beginning at 2 L and continued throughout the period of drainage. Despite greater volumes of colloid, however, significant NT-proBNP changes were still seen to arise, particularly among those patients with comorbid CCF. 

Congestive cardiac failure was the only independent predictor of significant NT-proBNP changes during LVP. Baseline NT-proBNP values were significantly higher in those with concurrent CCF (2626.93 pg/mL versus 528.68 pg/mL), and the mean decline in serum NT-proBNP during LVP was approximately 50 times greater in those with concurrent CCF than in those without (272.5 pg/mL versus 5.17 pg/mL). This implies a potentially higher risk of haemodynamic compromise and complications in these patients following LVP and suggests that cirrhotic patients with CCF may benefit from the earlier administration of greater volumes of supplemental albumin. Since NT-proBNP levels did not significantly decline from the time of albumin administration through to the end of drainage in all patient cohorts, this further validates the importance of colloid administration in abrogating any significant fluid shifts during LVP. 

Cirrhotic cardiomyopathy is an increasingly recognised entity which refers to the impaired cardiac contractility, systolic and diastolic dysfunction, and electromechanical abnormalities that can develop in patients with advanced cirrhosis [19, 20]. Its development can often be insidious, and it may not always be apparent on routine clinical assessment. Serum BNP and NT-proBNP are now recognised as valuable diagnostic and prognostic markers in the assessment of congestive cardiac failure. Their utility in the diagnosis of cirrhotic cardiomyopathy has also been examined. 

Pimenta and colleagues [21] demonstrated that patients with cardiac systolic dysfunction had higher baseline BNP values than those of cirrhotic patients without a diagnosis of heart failure. Similarly, Henriksen et al. [12] demonstrated independent correlations of several important markers of cardiac dysfunction (namely, greater QT interval, heart rate, and plasma volume) with higher levels of proBNP and BNP in cirrhotic patients not known to have CCF. They postulated that raised BNP and proBNP levels could serve to identify early cardiac dysfunction in patients with cirrhosis. This was seen in our cohort where significantly elevated NT-proBNP levels were found in three cirrhotic patients in whom a prior diagnosis of CCF had not been identified. On retrospective analysis, these patients were indeed found to have evidence of cardiac dysfunction on transthoracic echocardiography, which had been undertaken as part of clinical assessments into unrelated disorders. Routine baseline NT-proBNP measurements could therefore serve to detect those cirrhotic patients with unrecognised cardiomyopathy, as suggested by Sheer et al. [22], where serum NT-proBNP levels > 1000 pg/mL reliably distinguished between ascites due to CCF or cirrhosis. This would assist in the identification of cirrhotic patients at greatest risk of PCD following LVP.

Due to the small sample size in this study, these results should be interpreted with caution. This current analysis should be regarded as a pilot study which could provide the framework from which further trials could be undertaken in order to better delineate the utility of NT-proBNP. In particular, whether significant NT-proBNP changes correlate with more formal measures of PCD and longer-term outcomes such as ascites recurrence and mortality, and whether routine early administration of higher concentrations of supplemental albumin in cirrhotic patients with cardiac dysfunction can improve clinical outcomes.

## 5. Conclusions

In summary, significant NT-proBNP changes during LVP occur in patients with ascites secondary to cirrhosis but not malignancy. Congestive cardiac failure was an independent predictor of more significant NT-proBNP changes, further supporting the utility of natriuretic peptides in the diagnosis of cardiac dysfunction in patients with advanced cirrhosis, hence their predictive potential in identifying those at greatest risk of haemodynamic compromise and complications following LVP.

## Figures and Tables

**Table 1:** Baseline characteristics for patients with cirrhosis and malignancy.

Baseline characteristic	Cirrhosis (*n* = 19)	Malignancy (*n* = 15)	*P* value
Age (years)	59 (12.3)	62 (17.2)	0.62
Male gender	13 (68.4%)	7 (46.7%)	0.30
Primary cause of ascites	Etoh: 7 (37%), viral: 3 (15.8%), etoh + viral: 4 (21.1%), NASH: 4 (15.8), and other: 2 (10.6%).	Gynae: 7 (47%), GI: 3 (20%), and other: 5 (33%)	—
Child-Pugh score	B: 11 (58%), C: 8 (42%)	—	—
MELD score	16 (7)	—	—
Comorbid CCF/CRF/HT	**12 (63%)**	**3 (20%)**	**0.01**
Comorbid CCF	6 (32%)	1 (7%)	0.06
Diuretics	**12 (65.2%)**	**2 (13.3%)**	**<0.01**
Serum NT-proBNP (pg/mL)	1191.3 (1748.8)	567.3 (638.1)	0.16
Serum Na (mmol/L)	136 (4.7)	136 (5)	0.78
Serum creatinine (*μ*mol/L)	**102.6 (45.4)**	**77.0 (28.2)**	**0.05**
eGFR (mL/min)	64.6 (24.1)	76.7 (15.6)	0.09
Serum bilirubin (*μ*mol/L)	**58.2 (85.3)**	**11.6 (10.4)**	**0.03**
Serum albumin (g/L)	30.4 (5.3)	31.7 (4.7)	0.44
ALT (U/L)	37.0 (25.5)	24.2 (23.3)	0.14
AST (U/L)	72.6 (41.2)	46.6 (32.9)	0.06
GGT (U/L)	116. 6 (92.0)	116. 7 (190.7)	1.00
ALP (U/L)	158.0 (107.1)	155.5 (128.2)	0.95
INR	**1.56 (0.5)**	**1.05 (0.1)**	**<0.01**
Haemoglobin (g/L)	108.9 (20.4)	117.7 (20.7)	0.23
White cell count (×10^9^/L)	6.2 (3.7)	8.9 (4.6)	0.07
Platelet count (×10^9^/L)	**124.9 (66.0)**	**463.2 (193.3)**	**<0.01**
SAAG	**18.3 (4.4)**	**14.9 (4.1)**	**0.03**
Ascitic fluid protein (g/L)	**24.9 (8.0)**	**34.3 (12.9)**	**0.02**
Heart rate (beats/min)	**79.3 (14.7)**	**92.5 (15.3)**	**0.02**
Systolic blood pressure (mmHg)	114.7 (14.9)	124.8 (14.7)	0.06
Diastolic blood pressure (mmHg)	74.8 (19.0)	76.1 (7.0)	0.81
Total volume drained (Litres)	5.6 (2.1)	5.0 (1.9)	0.43

^*^All continuous variables have been expressed as a mean (SEM).

∗∗Renal failure is defined as eGFR < 60 mL/min.

MELD: model for end-stage liver disease; CCF: congestive cardiac failure; CRF: chronic renal failure; HT: hypertension; Na: sodium; eGFR: estimated glomerular filtration rate; ALT: alanine aminotransferase; AST: aspartate aminotransferase; GGT: gamma glutamate transferase; ALP: alkaline phosphatase; INR: international normalised ratio; SAAG: serum ascites albumin gradient.

**Table 2:** Clinical correlations with significant serum NT-proBNP changes at both time points.

2L prealbumin		End of drainage	
Clinical parameter	*P* value	Clinical parameter	*P* value
Age	0.06	Age	0.12
Gender	0.98	Gender	0.98
Child-Pugh score	0.38	Child-Pugh score	0.84
MELD	0.06	**MELD**	**0.05**
Cause of cirrhosis:		Cause of cirrhosis:	
Alcohol versus nonalcohol	0.13	Alcohol versus nonalcohol	0.41
History of		History of	
Varices	0.18	Varices	0.37
Previous SBP	0.83	Previous SBP	0.89
Previous HE	0.80	Previous HE	0.57
HCC	0.68	HCC	0.87
Comorbid:		Comorbid:	
CCF	0.12	** CCF**	**0.04**
CRF	0.35	CRF	0.36
HT	0.87	HT	0.66
Diuretics	0.62	Diuretics	0.11
Serum Na	0.84	Serum Na	0.83
Serum creatinine	0.21	Serum creatinine	0.43
eGFR	0.22	eGFR	0.46
Serum bilirubin	0.60	Serum bilirubin	0.24
Serum albumin	0.32	Serum albumin	0.93
ALT	0.58	ALT	0.44
AST	0.78	AST	0.77
**GGT**	**0.04**	GGT	0.34
ALP	0.10	ALP	0.15
Haemoglobin	0.58	Haemoglobin	0.78
White cell count	0.07	White cell count	0.26
Platelets	0.13	Platelets	0.31
INR	0.12	INR	0.23
**SAAG**	**0.05 **	SAAG	0.72
Ascitic protein	0.48	Ascitic protein	0.66
Ascitic albumin	0.47	Ascitic albumin	0.83
Baseline heart rate	0.56	Baseline heart rate	0.47
Baseline systolic BP	0.59	Baseline systolic BP	0.93
Baseline diastolic BP	0.59	Baseline diastolic BP	0.81
Total volume drained	0.79	Total volume drained	0.49
**Baseline NT-proBNP**	**<0.01**	**Baseline NT-proBNP**	**0.01 **

MELD: model for end-stage liver disease; HCC: hepatocellular carcinoma; CCF: congestive cardiac failure; CRF: chronic renal failure; HT: hypertension; Na: sodium; eGFR: estimated glomerular filtration rate; ALT: alanine aminotransferase; AST: aspartate aminotransferase; GGT: gamma glutamate transferase; ALP: alkaline phosphatase; INR: international normalised ratio; SAAG: serum ascites albumin gradient.

**Table 3:** Changes with colloid (albumin) administration.

	Change in NT-proBNP at 2L prealbumin	Change in NT-proBNP at end of drainage	Mean difference in NT-proBNP	*P* value	Additional fluid drained after 2 L	Concentrated albumin administered
All cirrhotics (*n* = 19)	−77.27 pg/mL	−94.29 pg/mL	−17.0 pg/mL	0.67	3.7 L	185 mls (37 grams)

Cirrhotics with concurrent CCF/CRF/HT (*n* = 12)	−88.63 pg/mL	−130.34 pg/mL	−41.7 pg/mL	0.32	4.1 L	205 mls (41 grams)

All values are expressed as a mean (SEM).

CCF: congestive cardiac failure; CRF: chronic renal failure; HT: hypertension.
